# Precision regenerative medicine

**DOI:** 10.1186/s13287-020-02092-w

**Published:** 2021-01-07

**Authors:** Amy L. Lightner, Timothy Chan

**Affiliations:** 1 Department of Colorectal Surgery, Digestive Disease Institute, Cleveland Clinic, 9500 Euclid Ave, Cleveland, OH 44195 USA; 2 Center for Immunotherapy and Precision Immuno-Oncology, Cleveland Clinic, Cleveland, OH USA; 3 National Center for Regenerative Medicine, Cleveland, OH USA

**Keywords:** Precision cell therapy, Individualized regenerative medicine, Mesenchymal stem cells

## Abstract

**Background:**

Precision or individualized medicine is increasingly pervasive across medical and surgical disciplines. However, the concept has not been introduced in regenerative medicine.

**Main body:**

Targeted and engineered cellular and acellular therapy, specific to individuals or disease states, could significantly improve the efficacy of these autologous therapies. Currently, generic mesenchymal stem cell therapy is being widely used across multiple pathologies, some of which may not respond well to mesenchymal stem cell therapy. Engineered cell therapy may be a way to address this generic one-size-fits-all approach.

**Conclusions:**

The future of regenerative medicine lies in the concept of precision cell-based therapy.

## Background

Every individual is unique. It should therefore be no surprise that each disease, tumor biology, and genome are specific to each individual, as is the response to medical therapy. This emerging reality is rapidly driving medicine toward “individualized” or “precision”-based medicine and surgery. Therapies are no longer designed and applied to all patients with a particular disease. Instead, therapy is targeted to the individual based on their genetic makeup, microbiome, or tumor biology.

## Main text

This concept of tailoring treatments to the individual patient has revolutionized cancer therapy and is gaining momentum in cardiovascular medicine, but has yet to gain widespread investigation in regenerative medicine and cellular therapy. In advanced colorectal cancer, KRAS mutations are an independent prognostic factor in treatment with cetuximab and wide-type KRAS is required for panitumumab efficacy [1, 2]. In breast cancer, the understanding of overexpression of HER2 new has completely changed treatment approaches with a monoclonal antibody against HER2 [3]. And, more recently, the treatment of melanoma has changed with immunotherapy and the understanding of mutations associated with acquired resistance to PD-1 blockade [4]. Similarly, cardiovascular medicine is becoming personalized. Patients with inactivating mutations in the gene encoding the trafficking protein PCSK9 are at a much lower risk for myocardial infarction, and patient response to warfarin and clopidogrel is affected by gene polymorphisms in VKORC1 and CYP2C19, respectively [5].

Regenerative medicine has lagged behind in the concept of individualized medicine, where genetic polymorphisms and individual response to therapeutics have remained largely unstudied within the context of improving outcomes with regenerative cellular and acellular therapies. This is an area of great potential for advancing regenerative medicine and surgery in two dramatic ways. First, understanding donor-to-donor variability of any autologous regenerative product is critical—the realization that a cell from one person is not the same as the same cell type from another donor suggests that donor screening may be an important first step in tailoring a therapeutic to a given disease. Second is the ability to engineer “smart” cellular or acellular therapeutics to target the disease being treated—one cell type does not fit all disease states.

The genome-wide project has eloquently displayed that every individual is unique down to our fundamental genetic makeup. This means that embryonic stem cells, induced pluripotent stem cells (iPS), and mesenchymal stem cells (MSCs) from any particular individual differ from the individual sitting next to them. Therefore, when investigating the efficacy of cell-based therapy, it is important to consider donor-to-donor variability and how this may affect cellular function as a therapeutic. For example, if an investigator wants to treat Crohn’s disease, an understanding that autologous MSCs from a Crohn’s patient are actually pro-inflammatory may lead the investigator to choose an allogeneic product instead. When searching for the optimal allogeneic MSC, finding a donor whose MSCs increase T regulatory cells and M2 macrophage polarization in vitro and in vivo may result in the best efficacy for Crohn’s. However, that same donor’s MSCs may not have the optimal anti-microbial characteristics needed for a cystic fibrosis therapeutic or the neovascular and remodeling properties desired for a myocardial infarction therapeutic. Similarly, each recipient of cell therapy is unique, and thus, their response to cell therapy is individualized. Genomic studies before and after cell delivery using sc-RNA and tissue RNA before and after cell therapy administration may shed light on how cell therapy works and who is an optimal responder.

An exciting potential of cell therapy is our ability to engineer cells, or alter their function, in a laboratory before use in clinical trials; with regard to scalability, this will likely be a more feasible approach using allogeneic cells. For example, MSCs have emerged as a promising therapeutic for immune-mediated and inflammatory disease due to their remarkable anti-inflammatory, immunosuppressive, and immunomodulatory properties carried out in both paracrine signaling and cell-to-cell contact mechanisms [6, 7]. However, of the over 900 pre-clinical and clinical trials in the last 10 years (source: http://www.clinicaltrials.gov), many have resulted in treatment failures or significant ranges in efficacy. A plausible explanation for these treatment failures is the expectation that one cell type, harvested from one particular location (i.e., MSCs from the bone marrow, adipose tissue, and umbilical tissue), from one particular donor, is expected to be effective in treating numerous diseases with varying pathophysiology. Rather than expecting one particular MSC product to be equally effective across multiple pathologies, MSCs could be optimized to treat any particular disease in a particular patient. MSCs can be primed with cytokines and growth factors, hypoxic conditions, pharmacological drugs, biomaterials, varying culture conditions, and diverse molecules to affect their immunomodulatory, regenerative, angiogenic, anti-apoptotic, and anti-scarring capabilities [8]. In addition, MSCs can also be engineered ex vivo through exogeneous delivery of DNA and RNA to alter gene expression, thereby improving in vivo therapeutic function. By upregulating or downregulating gene expression in MSCs or iPS cells, there is great potential to target tumor growth in malignancies or specific immunosuppressive effects needed for immune-mediated disease.

While developing “smart” cellular therapeutics through altering culture conditions or gene expression sounds promising, there are significant barriers for translation into human clinical trials. Cell manufacturing methods have to be replicated in good manufacturing practice (GMP) grade laboratories for the production of cellular products. Small and large animal models are also needed to test the safety and tumorigenic potential using good laboratory practice (GLP) grade facilities. While feasible, these steps are costly and time intensive which prohibit a rapid expansion of phase I, II, and III clinical trials with an eventual engineered off-the-shelf product. However, despite these limitations of scalable manufacturing, cost, delivery, and pace of clinical trials, we can begin to address personalization of cell therapy by directing each lot of cells toward a particular targeted use such as immune regulation, anti-inflammation, or osteogenesis.

## Conclusions

Despite these major limitations, emerging insight into the success of personalized or individualized medicine with oncology and cardiovascular medicine has highlighted the importance of individualizing therapy. In regenerative medicine, we need to apply these same principles to create a genre of “precision regenerative medicine.” This is our future, and where we may be able to achieve our greatest success.

## Data Availability

Not applicable
